# Natural products in osteoarthritis treatment: bridging basic research to clinical applications

**DOI:** 10.1186/s13020-024-00899-w

**Published:** 2024-02-15

**Authors:** Shunzheng Fang, Bin Zhang, Wei Xiang, Liujie Zheng, Xiaodong Wang, Song Li, Tongyi Zhang, Daibo Feng, Yunquan Gong, Jinhui Wu, Jing Yuan, Yaran Wu, Yizhen Zhu, Enli Liu, Zhenhong Ni

**Affiliations:** 1 School of Pharmacy, Medicinal Basic Research Innovation Center of Chronic Kidney Disease, Ministry of Education, Shanxi Medical University, Taiyuan, 030001 China; 2 State Key Laboratory of Trauma, Burns and Combined Injury, Department of Rehabilitation Medicine, Daping Hospital, Army Medical University, Chongqing, 400022 China; 3 Department of Wound Repair and Rehabilitation Medicine, Center of Bone Metabolism and Repair, Laboratory for Prevention and Rehabilitation of Training Injuries, State Key Laboratory of Trauma, Burns and Combined Injury, Trauma Center, Research Institute of Surgery, Daping Hospital, Army Medical University, Chongqing, 400022 China; 4 Rehabilitation Center, Key Specialty of Neck and Low Back Pain Rehabilitation, Strategic Support Force Xingcheng Special Duty Sanatorium, Liaoning, 125100 China; 5 Department of Orthopaedic Surgery, The Fourth Hospital of Wuhan, Wuhan, 430000 Hubei China

**Keywords:** Natural products, Osteoarthritis, Inflammation, NF-κB, Apoptosis

## Abstract

Osteoarthritis (OA) is the most prevalent degenerative musculoskeletal disease, severely impacting the function of patients and potentially leading to disability, especially among the elderly population. Natural products (NPs), obtained from components or metabolites of plants, animals, microorganisms etc., have gained significant attention as important conservative treatments for various diseases. Recently, NPs have been well studied in preclinical and clinical researches, showing promising potential in the treatment of OA. In this review, we summed up the main signaling pathways affected by NPs in OA treatment, including NF-κB, MAPKs, PI3K/AKT, SIRT1, and other pathways, which are related to inflammation, anabolism and catabolism, and cell death. In addition, we described the therapeutic effects of NPs in different OA animal models and the current clinical studies in OA patients. At last, we discussed the potential research directions including in-depth analysis of the mechanisms and new application strategies of NPs for the OA treatment, so as to promote the basic research and clinical transformation in the future. We hope that this review may allow us to get a better understanding about the potential bioeffects and mechanisms of NPs in OA therapy, and ultimately improve the effectiveness of NPs-based clinical conservative treatment for OA patients.

## Introduction

Osteoarthritis (OA) is a prevalent degenerative disease mainly resulting in cartilage damage and synovial inflammation, affecting a large number of individuals worldwide. The global number of OA patients has exceeded 250 million, while there is an increasing incidence rate each year, leading to a serious impact on individuals, healthcare systems, and the socioeconomic aspect of society. As lifestyles evolve and populations age, OA is becoming a major healthcare burden globally [1, 2]. It is the principal cause of motor disability, significantly impairing the quality of life [3]. Even though effective conservative methods can postpone OA progression, alleviate symptoms of joints, and ameliorate patients’ quality of life, the current clinical treatment outcomes for OA patients remain unsatisfactory. In addition, the underling mechanisms of OA are yet not completely revealed, which greatly hinders the application and optimization of relevant therapeutic approaches. Therefore, novel therapeutic strategies are needed to enhance the clinical treatment outcomes of OA.

Natural products (NPs), obtained from components or metabolites of plants, animals, microorganisms etc., are widely used as drugs in the present era [4]. Numerous studies have demonstrated that NPs offer potent therapeutic strategies for various diseases, including cancer, diabetes, and immune diseases [5–7]. NPs such as curcumin and Quercetin possess anti-inflammatory and antioxidant effects while exhibiting fewer toxic and side effects, thus improving patient compliance [5]. A multiplying body of studies demonstrate that NPs can effectively alleviate joint inflammation, restrain articular cartilage degradation, eventually slow up OA progression. These beneficial effects are attributed to their ability to inhibit inflammation, regulate anabolism and catabolism, and prevent chondrocyte death, among other mechanisms [8]. Thus far, the therapeutic effects of various types of NPs have been confirmed in OA animal models and clinical patients. With ongoing studies, the therapeutic potential of different NPs in OA treatment is being increasingly recognized, suggesting that NPs may play an important part in conservative treatments for OA in the future. In this review, we recap the present evidence on how NPs postpone the progression of OA. This compilation will provide a more thoroughly understanding of the potential value of NPs in OA therapy and facilitate the development of treatment options for OA patients.

## The underlying mechanisms of NPs in OA treatments

OA is a disease of the whole joint, whose pathologic changes include cartilage damage, bone, synovitis, subchondral bone sclerosis, and muscle atrophy [9]. Above them, cartilage damage is a key pathological change to induce pain, stiffness, functional limitation of OA patients. Recent studies reported that different types of NPs alleviate the process of OA via regulation inflammation, anabolism and catabolism, and cell death of cartilage. In this section, we will review the underlying mechanisms of NPs in OA treatments, including different signaling pathways affected by specific NPs.

## Inflammation signals that are regulated by NPs in OA

**Fig. 1:**
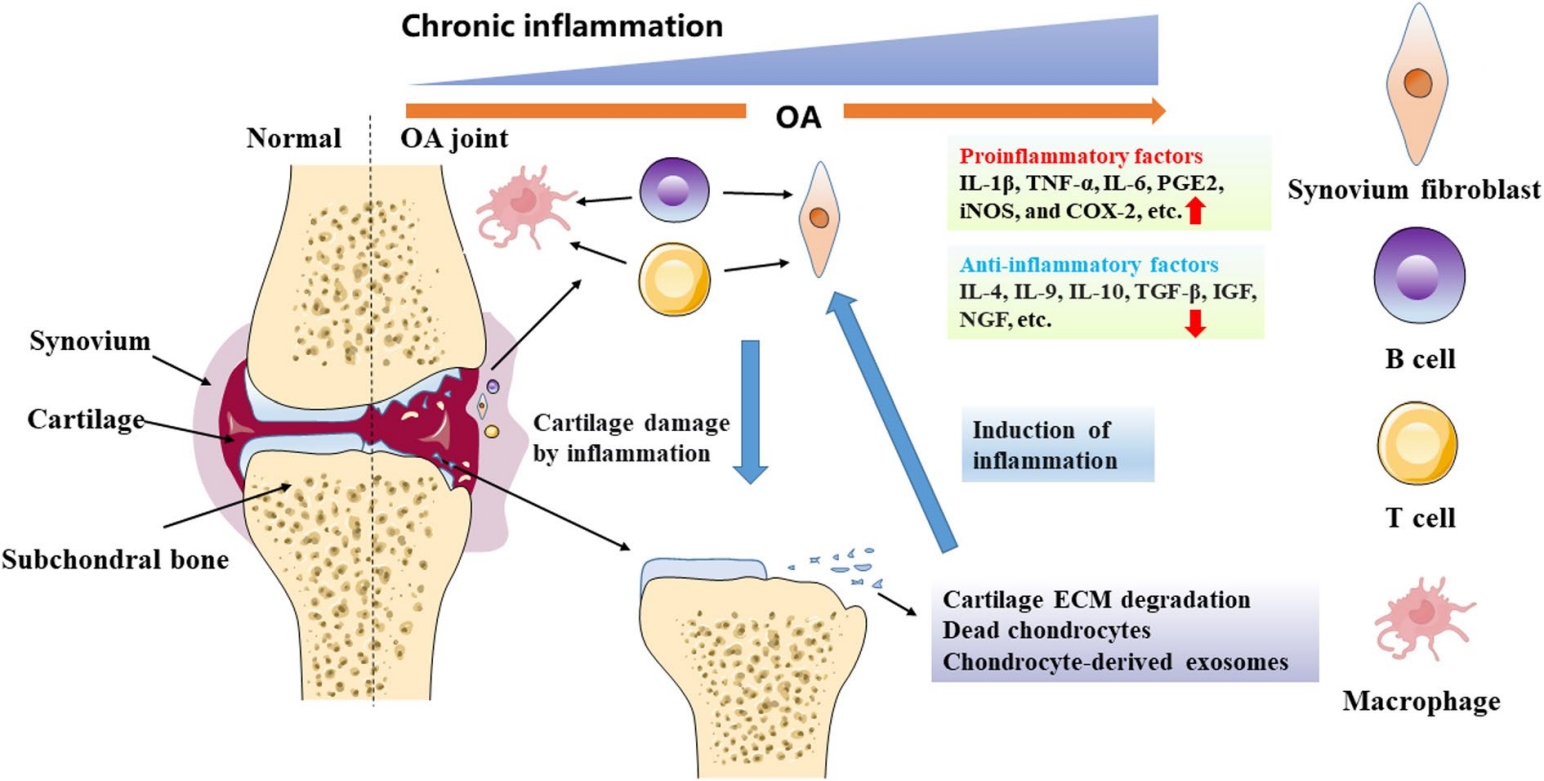
The inflammatory microenvironment of articular cartilage induced in OA. Chronic inflammation is an important pathological feature of OA and is a major factor in the destruction of cartilage; it also leads to persistent inflammation of the chondrocytes. Phagocytic clearance of chondrocyte degradation products, deceased chondrocytes, and chondrocyte-derived exosomes by synovial macrophages exacerbates the synovial inflammatory response. Inflammatory synovial cells generate pro-inflammatory factors, further intensifying chondrocyte degradation and establishing a vicious spiral. The activation of immune cells within the synovial membrane, such as B cells, T cells, and macrophages, increase the inflammatory level. Additionally, both synovium and cartilage produce anti-inflammatory cytokines to help modulate the inflammatory milieu

Inflammation closely involves the initiation of OA and affects chondrocyte metabolism during aging [10] (Fig. 1). The pro-inflammatory factors, such as IL-1β and IL-6, increase in joint fluid, synovium, and cartilage of OA [11, 12]. Furthermore, other factors like nitric oxide (NO), prostaglandin E2 (PGE2), and cyclooxygenase-2 (COX-2), mediating inflammation are increased in articular cartilage of OA [13, 14]. NO is a free radical that can induce cartilage destruction by increasing mitochondrial membrane potential (MMP) activity and declining production of aggrecan and collagen [15–17]. PGE2 is a critical lipid factor that acquires from arachidonic acid through catalyzed by COX enzymes and terminal PGE synthases [18]. PGE2 can promote the progression of OA, inhibit extracellular matrix synthesis and promote cartilage degradation [19]. COX-2 inhibitor has been wildly used in clinic for treatment of OA [20]. Therefore, inhibition of cartilage inflammation is a promising strategy for the OA treatment by NPs. We next review the typical signaling pathways about inflammation affected by NPs in OA.

### Nuclear factor-κB (NF-κB)

NF-κB is a rapidly inducible transcriptional regulator involved in inflammation and diverse cellular responses [21]. NF-κB pathway activation mainly involves three groups of proteins, which are NF-κB family (p50, p52, p65, c-Rel, and RelB), IκB family (IκB, IκBβ, IκBε, precursor proteins p100 and p105, Bcl-3, and IκBζ), and IKK complex (IKKα/IKK1, IKKβ/IKK2, and NF-κB essential modulator/IKKγ) [22]. IKK activation induces IκB phosphorylation and then degradation in the canonical pathway or turns p100 into p52 in the noncanonical pathway, causing transportation of phosphorylated NF-κB dimers into the nucleus [22]. NF-κB pathway is closely related to immune response. Cheng et al. recently reported that temporal dynamics of NF-κB activity plays an important part during the activation of latent enhancers of immune response genes [23]. NF-κB transcription factor is related to inflammation, anabolism, and catabolism of chondrocytes [24]. The categories of NPs that affect NF-κB in OA treatment are as follows.

#### IκB phosphorylation

Curcumin, a classic natural product of phenols, had demonstrated potent anti-inflammatory effects in chondrocytes of OA by suppressing IκB-α phosphorylation [25]. Through reducing phosphorylation of IκB-α, sinapic acid significantly inhibited the expression of IL-1α-induced IL-6 and TNF-α in human OA chondrocytes [26]. Sinomenine also had the ability to inhibit p-IκB-α at the protein level in IL-1β-treated chondrocytes [27]. Chrysin could inhibit phosphorylation of IκB-α in OA chondrocytes [28]. Asiatic acid reduced NF-κB activity via decreasing IκB-α phosphorylation in IL-1β-treated chondrocytes [29]. *Panax quinquefolium* saponin, an active ingredient of American ginseng, inhibited endoplasmic reticulum stress-activated inflammatory changes in OA cartilage by inhibiting phosphorylation of IκB-α [30].

#### IκB degradation

By suppressing IκB-α degradation, curcumin had potent anti-inflammatory effects in OA chondrocytes [25, 31]. Shikonin had anti-inflammatory effects on OA by suppressing IκB-α degradation in IL-1β-treated chondrocytes [32]. An extract of phenolic component, piperine, inhibited NF-κB by suppressing IκB-α degradation in the cytoplasm [33]. In chondrocytes, sinapic acid inhibited the expression of IL-1α-induced IL-6 and TNF-α via inhibiting IκB-α degradation [26]. Oleuropein, a phenolic component primary originated from olive, presented anti-inflammation function in human chondrocytes damage model by through degradation of IκB-α [34]. Polydatin, a natural extract belonging to resveratrol glucoside, had found to inhibit inflammation of OA by reducing cytoplasmic IκB-α degradation [35]. Eriodictyol, extracted from citrus fruits, rescued chondrocyte activity by reducing IκB-α degradation [36]. In OA chondrocytes, chrysin could inhibit the degradation of IκB-α [28]. β-Ecdysterone increased p-IκB-α degradation in inflammatory chondrocytes [37]. *Panax quinquefolium* saponin inhibited endoplasmic reticulum stress-activated inflammatory changes in OA cartilage by inhibiting IκB-α degradation [30]. Achyranthes bidentata blume (ABS) contains oleanane-type saponins considered as its main bioactive components. Xu et al. found that ABS had anti-inflammatory effects by inhibiting IκB-α degradation in IL-1β-treated chondrocytes [38].

#### P65 phosphorylation

Curcumin had potent anti-inflammatory effects in OA chondrocytes. It suppressed p65 phosphorylation [25]. Sinapic acid significantly inhibited the expression of IL-1α-induced IL-6 and TNF-α via inhibition of phosphorylation of p65 in human OA chondrocytes [26]. Danshensu, another product of phenols, inhibited the nuclear accumulation of p65 in osteoarthritic cartilages [39]. Salvianolic acid B dramatically reduced IL-1β-induced p65 phosphorylation in a mouse OA model [40]. Oleuropein presented anti-inflammatory function in human chondrocyte damage model by inactivating the phosphorylation of p65 [34]. Another terpenoid, ginsenoside Ro, belonging to the oleanolic acid-type ginsenosides, reduced apoptosis and inflammation in chondrocytes by in p65 phosphorylation [41, 42]. Glycyrrhizin inhibited the protein expression of HMGB1, TLR4 and p-p65 in cartilages of a rat OA model [43]. β-Ecdysterone also suppressed the phosphorylation of p65 in inflammatory chondrocytes [37].

#### P65 nuclear translocation

Curcumin could relieve OA chondrocyte inflammation by inhibiting p65 nuclear translocation [25, 31]. Recently, Chen et al. found that curcumin could bound to p65 by molecular docking and dynamic simulation studies [44]. Shikonin had anti-inflammatory effects in OA through the reduction of p65 in IL-1β-treated chondrocytes [32]. Moreover, moracin inhibited p65 nuclear translocation in chondrocytes of OA [45]. α-Mangostin, purified from mangosteen, protected articular chondrocytes from inflammation via inhibition of p65 in nuclear [46]. Liquiritigenin improved inflammation in IL-1β-treated chondrocytes by alleviating nuclear translocation and phosphorylation of p65 [47]. Myricetin down-regulated expression of generation of inflammatory mediators and cytokines by suppressing the expression and nuclear translocation of p65 in chondrocytes of OA [48]. Myricitrin had anti-inflammatory effects on mouse chondrocytes via reducing p65 nuclear translocation [49]. Piceatannol, a natural product from the family of stilbenes, had indicated to suppress the expression of nuclear p65 in IL-1β-stimulated chondrocytes [50]. Cryptotanshinone, another natural product, ameliorated the progression of OA by inhibiting nuclear translocation of p65 in chondrocytes [51]. Peiminine, belonging to alkaloids, had found to suppress IL-1β-induced expression of nuclear p65 in mouse chondrocytes [52]. Ligustilide, is an active component of Danggui, had an inhibitory effect on p65 nuclear dislocation in chondrocytes [53]. In summary, NF-κB signaling pathway is the most common pathway affected by NPs, including suppressing the expression and phosphorylation of cytoplasmic IκB and nuclear p65, increasing p-IκB degradation, and promoting p65 nuclear translocation.

### The mitogen activated protein kinases (MAPKs)

MAPKs are silk/threonine protein kinases that are widely distributed in eukaryotic cells. The MAPK signaling pathway may be activated by external signals or stimuli, including inflammatory cytokines, growth factors, bacterial complexes, and more. Following activation, the extracellular signals can be gradually amplified and transmitted to the nucleus. This is accompanied by the regulation of transcription factor activity and the corresponding gene regulation expression [54]. The MAPKs family includes extra-cellular signal-regulated kinase (ERK), p38, ERK3, and Jun N-terminal kinase (JNK), BMK1/ERK5, ERK27, ERK8 and NLK [55, 56]. And p38 can be classified into p38α, p38β, p38δ and p38γ isoforms. Among these, p38α and p38β are expressed in nearly all tissues and cells [57]. ERK includes ERK1-5 and ERK7/8 [58]. Three genes, jnk1, jnk2 and jnk3, encode the JNK proteins [59]. The p38MAPK signaling pathway can be activated by inflammatory factors and growth factors in the pathological process of OA, while inhibition of p-38 could ameliorate chondrocyte inflammation and OA progress [60, 61]. Inhibiting p-JNK and p-ERK can mitigate chondrocyte damage that is stimulated by IL-1β, decrease the production of inflammatory cytokines, and decelerate OA progression [62]. MAPKs display close associations with chondrocyte inflammation, anabolism, and catabolism. Different types of NPs could inhibit inflammation of OA chondrocytes via MAPKs. (1) Flavonoids. Wogonin inhibited inflammatory changes in OA cartilage by activating Nrf2 through phosphorylation of ERK1/2 in OA chondrocytes [63]. Liquiritigenin had demonstrated potent anti-inflammatory effects in OA chondrocytes by inhibiting IL-1β-induced activation of MAPKs, including down-regulation of p-ERK, p-p38, and p-JNK [47]. In chondrocytes affected by OA, Astragalin was found to inhibit the phosphorylation of ERK, JNK, and p38 [64]. (2) Quinones. Cryptotanshinone suppressed the phosphorylation of ERK, JNK, and p38 in chondrocytes in OA [51]. (3) Phenols. Isorhapontigenin (ISO) down-regulated p-ERK and p-p38 protein levels in cartilage, thereby influencing the progression of OA [65]. By suppressing IL-1β-induced activation of MAPKs, including downregulation of JNK, caffeic acid had shown potent anti-inflammatory effects in OA chondrocytes [66]. (4) Phenylpropanoids. Schisantherin A reduced the levels of p-ERK, p-p38, and p-JNK proteins in cartilage, thereby affecting the progression of osteoarthritis [67]. (5) Terpenoids. Echinocystic Acid had shown to have potent anti-inflammatory effects in OA chondrocytes by suppressing p-ERK, p-JNK, and p-p38 [68]. The current studies mainly tested the expression of MAPKs affected by NPs, but more detailed mechanisms like the how NPs influence MAPKs are badly needed to be explored in the future study.

### Phosphatidylinositol 3-kinase/protein kinase B (PI3K/AKT)

PI3K is a catalytic signaling protein that is widely present in various cells of the body and is involved in cell processes such as proliferation, migration, and apoptosis [69]. AKT is divided into three isoforms, PKB-α, β and γ, and is the major downstream effector molecule of PI3K. Activation of the PI3K/Akt pathway can gradually phosphorylate the Akt protein, and Akt can act on a variety of protein substrates and change their state after activation. It promotes the regulation of cell activity, inhibits cell apoptosis, regulates energy metabolism and other biological effects [70]. PI3K/AKT have a crucial function in the proliferation and growth of skeletal system cells [71]. Xue et al. found that inhibition of PI3K/AKT/MTR signaling reduced inflammatory responses and promoted autophagy in chondrocytes [72]. There are still many different types of NPs that have significant effects on this pathway. (1) Flavonoids. For instance, Farrerol demonstrated anti-inflammatory properties in OA by suppressing the production of p-PI3K and p-AKT in OA chondrocytes [73]. Myricetin could protect cartilage and alleviate the progression of OA via inhibition of PI3K/AKT-mediated Nrf2/HO-1 signaling pathway in chondrocytes [48]. (2) Phenols. Astilbin could inhibit inflammation in IL-1β-treated chondrocytes by downregulating the signaling pathway of PI3K/AKT [74]. Oroxin B reduced PI3K and AKT phosphorylation and reduces inflammation in chondrocytes [75]. Urolithin A inhibited inflammation in IL-1β-treated chondrocytes by downregulating p-PI3K and p-AKT [76]. Leonurine was found to suppress the phosphorylation of PI3K and AKT in chondrocytes affected by OA [77]. (3) Terpenoids. By inhibiting IL-1β-induced activation of p-PI3K, p-AKT and p-mTOR, artemisinin had demonstrated potent anti-inflammatory effects in OA chondrocytes [78]. PI3K/AKT pathway not only significant in OA chondrocytes, but also synovial macrophages. Zheng et al. found that mechanical loading mitigated synovial inflammation and changed the ratio of M1 and M2 macrophages by PI3K/AKT pathway [79]. However, whether NPs could regulate the inflammation of OA by PI3K/AKT pathway in macrophages is not clear.

### Silent information regulator factor 2-related enzyme 1 (SIRT1)

The sirtuins belong to deacetylase, SIRT1-7, with the ability to prevent metabolic diseases and aging [80]. SIRT1 can target several transcriptional proteins related to DNA repair, metabolism, inflammation, cancer, etc., such as p53, NF-κB and Forkhead [81, 82]. In OA, SIRT1 exerts anti-inflammation and anti-catabolic ability in OA. Batshon et al. found that the increased levels of serum N-terminal polypeptide/C-terminal fragment SIRT1 ratio correlated with moderate OA patients, which may induce prolonged inflammatory insult [83]. Disruption of Sirt1 in chondrocytes aggravated progression of OA [84]. The activation of SIRT1 attenuated OA development though inhibiting synovitis [85]. There are limited literatures about different NPs’ treatment of OA via SIRT1 signaling pathway. (1) Ketones. In addition, safranal could potentially prevent OA development via up-regulating expression of SIRT1 [86]. (2) Phenols. Resveratrol, a natural activator of SIRT1, could inhibit OA disease progression, which has been well reviewed by Deng and his colleagues [87]. (3) Flavonoids. Fisetin suppressed the production of pro-inflammatory factors like NO, PGE2, IL-6 and TNF-α through increasing expression and activity of SIRT1 in on human OA chondrocytes [88]. Since SIRT1 pathway is important in the OA progress, more studies are needed to explore this pathway affected by NPs during the treatment of OA in the future.

### Other signaling pathways

Beside the above typical signaling pathway related to inflammation, there are limited studies reported that other pathways were affected by NPs in the treatment of OA. (1) Terpenoids. For example, triptolide exerted its anti-inflammatory effect on OA by downregulating hsa-miR-20b, whose target gene is NLRP3 [89]. (2) Flavonoids. Besides, quercetin reduced IL-1β-stimulated inflammation of rat chondrocytes via inhibition of IRAK1/NLRP3 pathway [90]. Thus, NPs could regulate the inflammation of OA via epigenetics. Recently, Tan et al. reviewed that NPs can regulate the glycolytic pathway in the treatment of OA [91]. During the treatment of OA, we speculate that more signaling pathways regulated by virous NPs will be found and studied in the future.

**Fig. 2:**
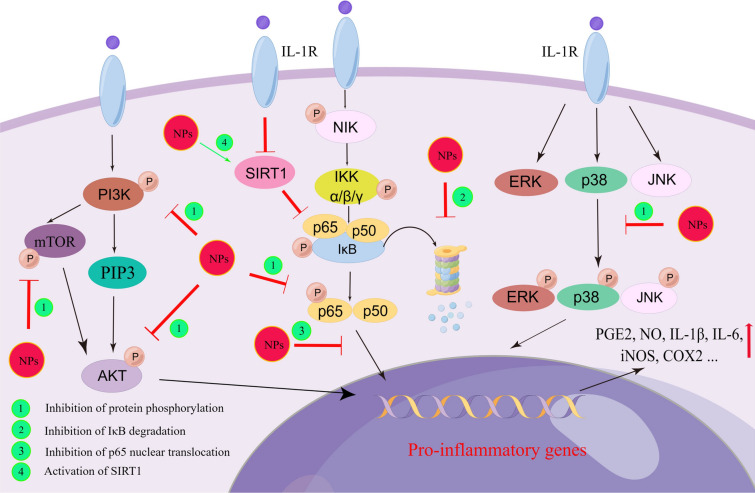
The main inflammatory signaling pathways affected by NPs during OA treatment. NPs inhibit NF-κB pathway by inhibiting IκB degradation and p65 translocation into the nucleus, as well as phosphorylation of p65. NPs inhibit the phosphorylation of PI3K, mTOR and AKT in PI3K/AKT pathway. NPs also prevent the phosphorylation of p38, JNK and ERK, thereby blocking the activation of MAPKs-related pathways. In addition, NPs can inhibit the SIRT1-mediated activation of NF-κB pathway, thereby reducing the IL-1β-induced expression of inflammatory mediators in chondrocytes. The inhibition of above signaling pathways by NPs decrease the production of several inflammatory factors, including PGE2, NO, IL-1β, IL-6, iNOS, COX2, etc.

In brief, several signaling pathways that regulate inflammation can be modulated by NPs (Fig. 2). So far, most studies have focused on exploring the anti-inflammatory mechanism of NPs in chondrocytes. Since osteoarthritis is a disease of the whole joint, further studies may need to concentrate on other types of cells within the joint, including but not limited to synovial fibroblasts, macrophages, osteoblasts, and adipocytes in the infrapatellar fat pad, to gain a comprehensive understanding of the potential and mechanism of NPs in OA treatment.

## Anabolism and catabolism that are regulated by NPs in OA

**Fig. 3:**
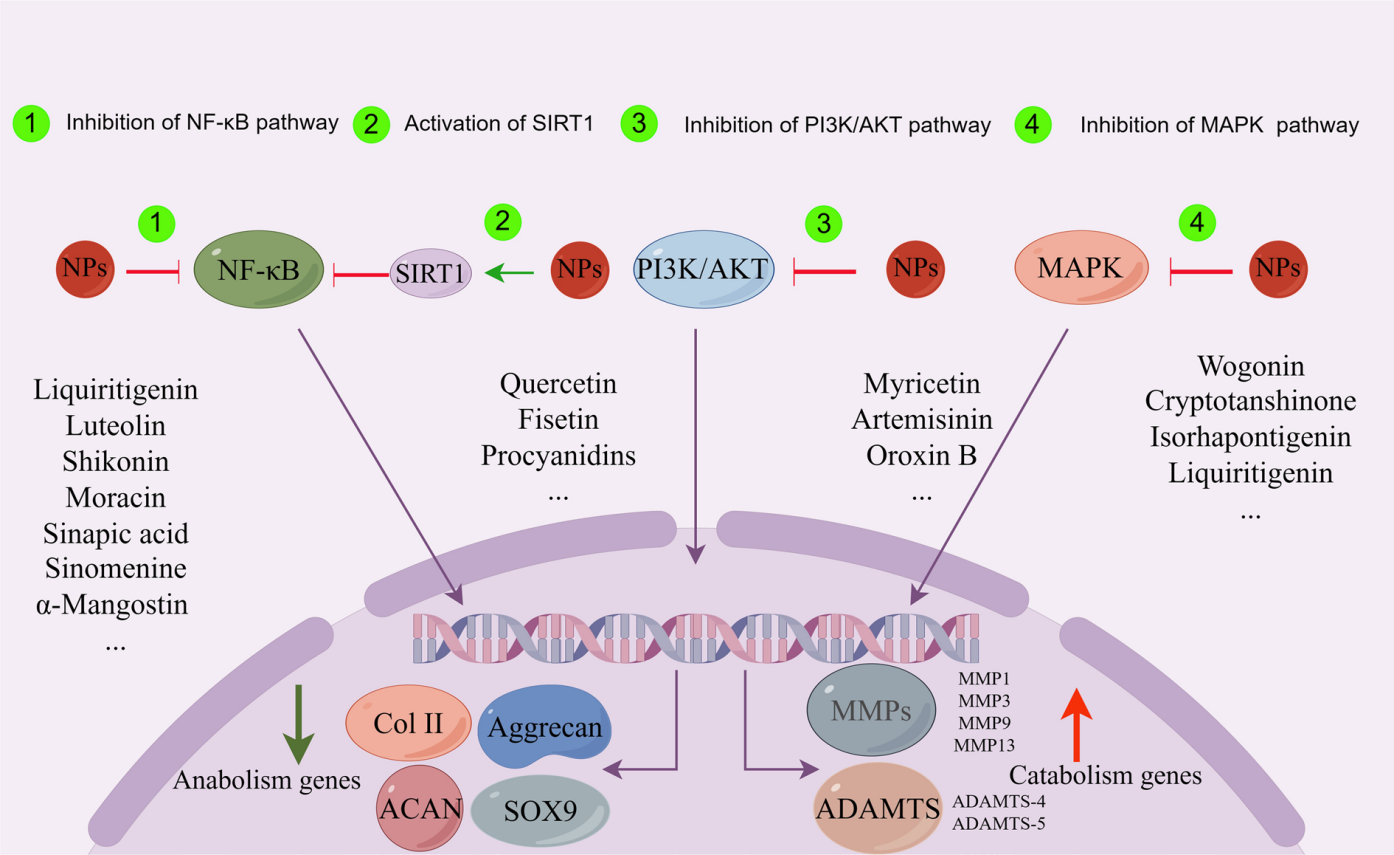
The main signaling pathways in catabolism and anabolism of cartilage affected by different types of NPs. The regulation of anabolic and catabolic genes in IL-1β-treated chondrocytes by NPs involved multiple signaling pathways, including MAPK, PI3K/AKT, SIRT1, and NF-κB. By affecting the above signaling pathways, NPs promote the up-regulation of anabolic substances such as aggrecan, Col II, ACAN and SOX9, and inhibit the production of catabolic substances such as ADAMTS and MMPs

Anabolism and catabolism are crucial for maintaining chondrocyte homeostasis and exert a key influence on the pathogenesis of OA [92]. Imbalances between catabolic and anabolic activities can result in chondrocyte degeneration [93]. Various factors, including mechanical loading, aging and inflammation, have been shown to influence the catabolic and anabolic processes of chondrocytes, thereby impacting the progression of OA [94, 95]. The expression of anabolic genes such as SOX9, collagen II, and aggrecan decrease in senescent chondrocytes, whereas the expression of catabolic genes such as matrix metalloproteinase-1 (MMP-1), MMP-9, MMP-13, thrombospondin motifs 5 (ADAMTS-5), matrix metalloproteinase 1 (TIMP-1), TIMP-2 and C-telopeptide of type II collagen (CTX-II) increase [93, 96]. Additionally, the expression of collagen II, SOX9, and aggrecan exhibit a notable decrease in chondrocytes stimulated by IL-1β [97]. Age-related changes in pathways such as NF-κB, MAPK, AKT, and SIRT1 can affect chondrocyte metabolism in OA [98]. Targeting the signaling pathways involved in catabolism and anabolism represents a viable strategy for the therapeutic management of OA (Fig. 3). NPs have demonstrated the capacity to modulate chondrocyte anabolism and catabolism, offering a promising strategy to preserve cartilage quality and delay OA progression.

### NF-κB

NF-κB signaling pathway is closely related to anabolism and catabolism, especially in cartilage tissue of OA. In the process of OA, excessive mechanical loading promotes cartilage degeneration by activating NF-κB pathway [94], while inhibiting NF-κB protects cartilage from degeneration and degradation [99]. Many NPs, belonging to phenols, flavonoids, terpenoids, stilbenes, and others, can affect NF-κB pathway in several ways.

#### IκB phosphorylation

Curcumin inhibited MMP-13 and increased the production of SOX9 by reducing IκB-α phosphorylation [25]. Asiatic acid down-regulated IκB-α phosphorylation, which suppressed the expression of MMP-13 and collagen type X [29]. Morroniside could improve cartilage matrix synthesis by increasing collagen type II expression, related to inhibition of the IκB-α phosphorylation [100].

#### IκB degradation

Shikonin had chondro-protective effects, including reduction of MMP-1, MMP-3 and MMP-13 in IL-1β stimulated chondrocytes, by inhibiting IκB-α degradation [32]. Curcumin inhibited MMP-13 and increased the production of type II collagen through the degradation of IκB-α [101]. Chlorogenic acid inhibited the IL-1β-induced degradation of IκB-α in chondrocytes, which inhibited the expression MMPs while increases TIMP-1 expression [102]. The expression of MMP-13 and ADAMTS-5 could be reversed by salvianolic acid B in a mouse OA model via inhibiting degradation of IκB [40]. Another production of phenols, emodin, ameliorated cartilage degradation in OA by inhibiting IκB-α degradation [103]. Besides, aqueous extract of *Anthriscus sylvestris*, major antioxidant components of ethanol extracts, suppressed expression of nitrite, iNOS, PGE2, COX-2, MMP-3, MMP-13, and ADAMTS-4 in IL-1β-treated chondrocytes, which related to suppression of IκB-α degradation [104].

#### P65 phosphorylation

Chemically modified curcumin promoted collagen 2a1 and suppressed MMP-3, runt-related transcription factor 2 and cleaved caspase-3 by inhibiting phosphorylation of p65 in chondrocytes [105]. Sinapic acid reversed the degradation of type II collage and aggrecan, as well as MMP-9, MMP-13, and ADAMTS-5 by suppressing p65 phosphorylation in OA chondrocytes [26]. Danshensu reduced the expression and activity of MMP-3 and MMP-13 by inhibited p65 phosphorylation in osteoarthritic cartilages [39]. Liquiritigenin inhibited cartilage matrix degradation, including suppressing MMP-3, MMP-13, ADAMTS-4 and ADAMTS-5, by alleviating phosphorylation of p65 [47]. Luteolin reversed the degradation of collagen type II by suppressing the phosphorylation of p65 in IL-1β-induced chondrocytes [106]. Aucubin inhibited gene and protein expression of MMP-3, MMP-9, and MMP-13 of chondrocytes via repressing phosphorylation of p65 [107]. The expression of MMP-3, MMP-9, and MMP-13, and the degradation of type II collagen and aggrecan could be reversed by α-Mangostin through inhibiting p65 phosphorylation [46]. β-Ecdysterone prevented matrix degradation via reducing MMP-3 and MMP-9, which was related to p65 phosphorylation of chondrocytes [37].

#### P65 nuclear translocation

Danshen reduced the expression and activity of MMP-9 and MMP-13, while it promoted the expression of TIMP-1 and TIMP-2 via inhibiting the nuclear translocation of p65 [108]. Salidroside upregulated the levels of type II collagen and aggrecan, and downregulated MMP-13 via suppressing the expression of nuclear p65 in chondrocytes of OA rats [109]. Hyperoside ameliorated the Extracellular matrix (ECM) degradation during the progression of OA via reducing nuclear translocation of p65 in chondrocytes [110]. Myricetin down-regulated expression of MMP-13 and ADAMTS-5, which caused cartilaginous degradation, via inhibiting the expression and nuclear translocation of p65 in chondrocytes of OA [48]. The expression of MMP-13, MMP-3 and ADAMTS-5 and degradation of collagen-II could be reversed by myricitrin via reducing nuclear translocation of p65 [49]. Using mice model of destabilization of the medial meniscus, Hu et al. found that loganin increased collagen type II and decreased MMP-3, MMP-13, and collagen type 10 in cartilage by reducing nuclear translocation of p65 in chondrocytes during OA development [111]. In addition, sauchinone (SAU), a compound extracted from saururus chinensis, was able to protect chondrocytes from hypertrophy via down-regulating expression of nuclear p65 [112]. Piceatannol inhibited ECM degradation by inhibiting nuclear p65 in human OA chondrocytes [50]. Ligustilide inhibited the IL-1β-stimulated expression of MMP-3, ADAMTS-5 by inhibition of p65 nuclear dislocation in chondrocytes [53]. In summary, NPs influence anabolism and catabolism of cartilage, including inhibiting catabolic genes and increasing anabolic genes mainly via affecting IκB phosphorylation, IκB degradation, p65 nuclear translocation and p65 phosphorylation.

### MAPKs

MAPKs are important upstream signaling pathways in the cartilage metabolism [113]. The activation of ERK1/2 can induce the synthesis of MMP-3 [114]. Besides, inhibition of p38, p44/42 and Src family alleviated cartilage degradation by blocking MMP synthesis and activity, while only p44/42 was essential for aggrecan degradation [113]. 1. Flavonoids. Wogonin inhibited MMP secretion through activation of Nrf2 via phosphorylation of ERK1/2 in OA chondrocytes [63]. 2. Quinones. Cryptotanshinone inhibited the IL-1β-induced expression of MMP-3, ADAMTS-5, and MMP-13 in OA chondrocytes by suppressing p-ERK, p-JNK, and p-p38 [51]. 3. Phenols. ISO increased the expression of collagen type II and decreased the expression of MMP-3, MMP-13 and collagen type I in cartilage [65]. This was accomplished by reducing the expression of p-ERK and p-p38 in chondrocytes during the development of OA [65]. Caffeic acid could prevent IL-1β-induced degradation of collagen II and aggrecan in chondrocytes through suppression of JNK [66]. 4. Phenylpropanoids. Schisantherin A suppressed the IL-1β-stimulated expression of MMP-1, MMP-3, and MMP-13 in chondrocytes affected by osteoarthritis by inhibiting p-ERK, p-JNK, and p-p38 [67]. 5. Terpenoids. During the development of OA, Echinocystic Acid reduced the expression of MMP-3 by down-regulating the expression of p-ERK, p-JNK, and p-p38 in chondrocytes [68]. In a word, MAPKs pathways not only regulates inflammation during OA treatment of different NPs, but anabolism and catabolism in chondrocyte. The distinct mechanisms underlying inflammation and metabolism warrant further examination.

### PI3K/AKT

PI3K/AKT pathway is essential for both normal metabolism of joint tissues and development of OA [71]. The activation of AKT promoted synthesis of collagen II in cartilage anabolism, inhibiting cartilage degradation of OA [115]. However, in the IL-1β-induced OA model, selective inhibitor blockade of Akt pathway reduced expression of MMPs in chondrocytes cartilage collagen release [116]. Many NPs regulate cartilage anabolism through inhibiting PI3K/AKT pathway. 1. Flavonoids. Myricetin increased the expression of collagen II and aggrecan while suppressed the expression of MMP-13 and ADAMTS-5 in chondrocytes stimulated by IL-1β, via inhibiting the Nrf2/HO-1 signaling pathway stimulated by PI3K/AKT [48]. 2. Phenols. Oroxin B reduced the expression of MMP-3 and MMP-13 by downregulating the production of p-PI3K and p-AKT in chondrocytes [75]. Daurisoline, an isoquinoline alkaloid, alleviated the HO-induced high expression of MMP-3 and MMP-13 and degradation of type II collagen [117]. Urolithin A downregulated the production of p-PI3K and p-AKT in chondrocytes and reduced the expression of ADAMTS-5 and MMP-13 [76]. Mulberroside A, a natural bioactive with anolide, affected anabolic and catabolic-related proteins like aggrecan and MMP-13 by inhibition of PI3K-AKT-mTOR pathway in OA mice model [118]. Leonurine suppressed IL-1β-stimulated expression of ADAMTS-5 and MMP-13 in OA chondrocytes through inhibiting of p-PI3K and p-AKT [77]. 3. Terpenoids. Artemisinin inhibited the expression of MMP-3, MMP-13, and ADAMTS-5 in cartilage by suppressing the PI3K/AKT/mTOR signaling pathway to reduce cartilage degradation [78]. Since simply activation or inhibition of PI3K/AKT/mTOR signaling maybe a double-edged sword during the treatment of OA [71], more studies are needed to explore the detailed bioeffects and mechanisms of NPs on PI3K-AKT pathway to protect against OA.

### SIRT1

SIRT1 activity is beneficial to extracellular matrix expression during and cartilage development by using human embryonic stem cells, which was related to ARID5B-SIRT1 interaction [119]. In addition, overexpression of SIRT1 in IL-1β-stimulated chondrocytes has been indicated to inhibit the upregulation of MMP-1, MMP-2, MMP-9, MMP-13, and ADAMTS-5 through the NF-κB pathway [120]. SIRT1 is also a major deacetylase responsible for SOX9 deacetylation in human chondrocytes [121]. SIRT1 activator SRT1720 increased collagen type II alpha 1 and aggrecan in cartilage, attenuating development of OA [85]. Limited studies reported that SIRT1 can be regulated by NPs during treatment of OA. 1. Terpenoids. Bilobalide, extracted from Gingko biloba, promoted extracellular matrix synthesis and inhibited proteolytic enzyme activities through activation of the AMPK-SIRT1 pathway [122]. 2. Phenols. Procyanidins, a grape seed extract belonging to phenols, attenuated apoptosis and senescence of chondrocytes by inhibition of dipeptidase-4, which depended on activation of SIRT1 [123]. 3. Flavonoids. Quercetin attenuated pro-catabolic responses in rats’ knee chondrocyte, including down-regulation of MMP-3 and MMP-13 expression [124]. Fisetin inhibited degradation of SOX9, aggrecan and collagen-II in IL-1β-stimulated chondrocytes by increasing protein and activation of SIRT1 [88]. More relevant studies are needed to explore the underlying mechanisms.

## NPs regulate cell death in OA

**Fig. 4:**
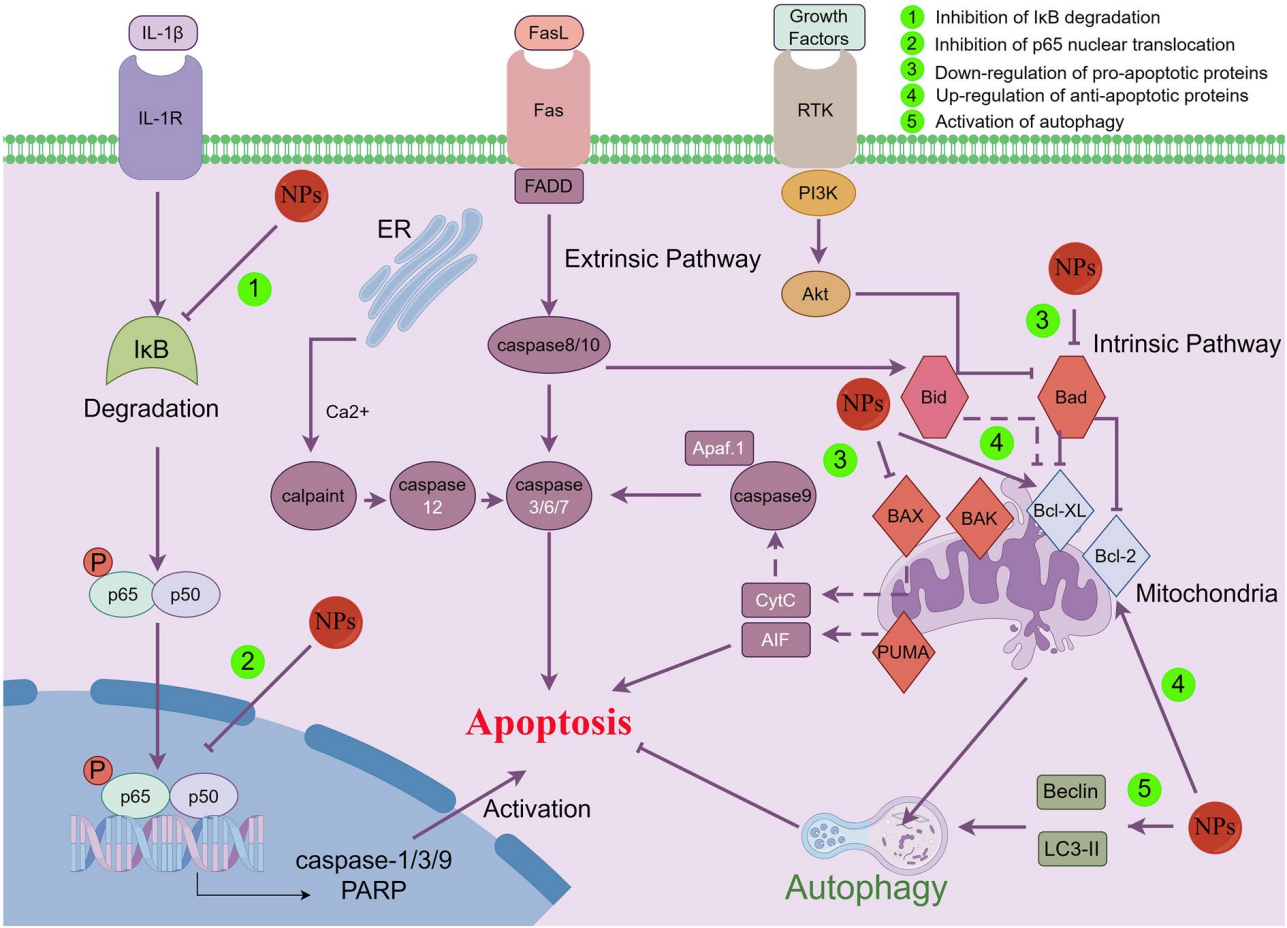
Apoptosis-related signaling pathways in chondrocyte influenced by NPs. NPs mainly affect apoptosis through intrinsic rather than extrinsic pathway, including up-regulation of anti-apoptotic proteins and down-regulation of pro-apoptotic proteins. NPs can reduce the production pro-apoptosis-related genes such as cleaved-caspase via inhibiting IL-1β-induced phosphorylation of IκB and nuclear translocation of p65 in chondrocyte. In addition, NPs enhance autophagy and further protect chondrocytes from IL-1β-induced apoptosis in IL-1β-treated chondrocytes

Cell death is a pivotal physiological phenomenon in living organisms [125], whose types include apoptosis, pyroptosis, and ferroptosis [126–128]. During the progress of OA, chondrocyte degeneration is closely linked with pyroptosis and apoptosis [129, 130]. Apoptosis is the most common type of chondrocyte death in studies of NPs for OA treatment (Fig. 4). Apoptosis involves external and internal pathways, including the TNF and fas ligand mediated external pathway, the Bcl-2 and Bax mediated internal mitochondrial pathway, and the Bcl-2 and caspase-8 mediated endoplasmic reticulum stress pathway [131]. Reducing chondrocyte apoptosis could prevent articular cartilage from destruction [132]. Inhibition of the NLRP3/caspase-1 pathway has been shown to suppress apoptosis of chondrocyte in knee OA [133], while activation of the NF-κB signaling pathway can suppress chondrocyte apoptosis in OA [134]. Previous studies showed the anti-apoptotic properties of several NPs on chondrocytes in OA. (1) Phenols. Paeonol attenuated apoptosis in OA chondrocytes [135]. Wogonin protected against apoptosis in IL-1β-induced human OA chondrocytes by suppressing ROS production [63]. Curcumin promoted chondrocyte autophagy by upregulating Beclin-1 and ATG5 gene expression and inhibited chondrocyte apoptosis via inhibition of p65 activation [44]. (2) Terpenoids. Ginsenoside Ro could inhibit chondrocyte apoptosis by promoting the expression of Bcl-XL and PCNA, inhibiting the expression of Bad and Bax, and reducing caspase-3 activity [42]. Tanshinone I inhibited apoptosis in CHON-001 cells within an IL-1β-induced cellular model [136]. Genistein alleviated apoptosis stimulated by IL-1β by inhibiting the production of caspase 3 in IL-1β-treated OA chondrocytes [137]. Morroniside inhibited pyroptosis and apoptosis of chondrocyte by inhibiting NF-κB signaling pathway [100]. (3) Steroids. β-Ecdysterone inhibited apoptosis in IL-1β-treated chondrocytes by modulating the expression of apoptotic markers, including anti-apoptotic protein Bcl-XL and pro-apoptotic protein Bax [37]. (4) Mixture. Danshen, a member of the Lamiaceae family, alleviated cartilage degeneration via increasing the expression of Bcl-2 and reducing the expression of Bax [108]. Achyranthes bidentata saponins suppressed chondrocyte apoptosis through promoting anti-apoptotic protein Bcl-xL and suppressing caspase-3 activation, as well as inhibiting the Bad and Bax expression [38]. These compounds exert their effects by modulating the production of apoptotic and anti-apoptotic proteins, inhibiting activity of caspases, and suppressing ROS production. Considering that other types of cell death like ferroptosis and copper death have been well studied recently, it is imperative to concentrate on the role of NPs in those cell death during the treatment of OA.

## The therapeutic effect of NPs in OA animal models

The therapeutic effect of NPs on OA has been investigated in lots of animal models, such as rats, mice, and rabbits, using surgical or drug-induced approaches. Surgically induced animal models include destabilization of the medial meniscus (DMM), anterior cruciate ligament transection combined with medial menisci resection (ACLT + MMx), and ACLT. Additionally, a drug-induced animal model is achieved through intra-articular injection of mono-iodoacetate (MIA). These models could uncover the pathological changes and underlying mechanisms of NPs’ effect on OA treatment (Table 1), including but not limited to cartilage matrix degradation, osteophyte formation, and cartilage calcification.

**Table 1:** Effects of NPs on animal OA models

List	Compound	Source	Route of administration	Animal model	Biological effect	Mechanism	References
1	Chlorogenic acid	Coffee and beans	Intra-articular injection	Rabbit OA model induced by ACLT	Cartilage degradation↓	Cartilage catabolic genes: MMP-13, MMP-1, and MMP-3↓, TIMP-1↑ Signal transduction pathway: NF-κB↓	[102]
2	Detoxicated Fuzi	*Aconitum carmichaeli*	Oral administration	Rat OA model induced by MIA	Osteolysis↓ Swelling↓ Irregularity of cartilage surface↓ Bone mineral density↑	–	[149]
3	EU aqueous extract	EU	Gavage	Rat OA model induced by modified Hulth technique	Osteophyte↓ Incomplete and thickening articular surface↓	Inflammatory factors: IL-1β and IL-6↓ Cartilage catabolic gene: MMP-3↓ Signal transduction pathway: p-AKT↓	[150]
4	Ginsenoside Rb1	Chinese herb *Panax ginseng*	Intra-articular injection	Rat OA model induced by ACLT(ROMIBA)	Cartilage damage↓	Signal transduction pathways: JAG1 and Notch1↓ Cartilage catabolic gene: MMP-13↓ Cartilage anabolic gene: collagen II↑	[142]
5	Pomegranate Fruit Extract	*Punicagranatum* L, Punicaceae	Oral consumption	Rabbit OA model induced by ACLT	Cartilage damage↓	Cartilage catabolic genes: MMP-3, MMP-9↓ Inflammatory factor: PGE 2 ↓ Cartilage anabolic genes: ACAN, COL2A1↓ Apoptosis related factor: caspase-3↓ Signal transduction pathway: PARP p85↓	[151]
6	Ginsenoside Rg1	Ginseng	Gavage	ROMIBA	Cartilage erosion↓ Abrasion of the articular surface↓	Inflammatory factors: COX-2, PGE 2↓ Cartilage catabolic gene: MMP-13↓ Cartilage anabolic genes: type II collagen, aggrecan↑	[144]
7	Fisetin	Mangoes, grapes, apples	Gavage	Mouse OA model induced by DMM(MOMIBD)	Subchondral bone plate↓ Synovitis↓	Cartilage anabolic gene: proteoglycan↑	[88]
8	Salvianolic acid B	*Salvia miltiorrhiza* Bunge	Intraperitoneal-injection	MOMIBD	Cartilage matrix degradation↓ Destruction of articular cartilage↓ Cartilage degradation↓	–	[40]
9	Cryptotanshinone	*Salvia miltiorrhiza* Bunge	Gavage	Mouse OA model	Destruction of the cartilage surface Cartilage erosion↓ Proteoglycan loss↓	Cartilage anabolic gene: proteoglycan↑	[51]
10	α-Mangostin	*Garcinia mangostana*-L.	Intraperitoneal injection	Rat OA model induced by DMM	Decrease of chondrocyte↓ Articular cartilage thickness↑	–	[46]
11	Piceatannol	Euphorbia lagascae	Gavage	MOMIBD	Destruction of cartilage surface↓ Cartilage calcification↓ Osteophyte↓ Synovitis↓	–	[50]
12	Extract of *Anthriscus sylvestris* leaves	*Anthriscus sylvestris*	Oral admin-Istration	Rat model of OA induced by DMM	Proteoglycan depletion↓ Destruction of cartilage surface↓ Cartilage erosion↓	Cartilage anabolic gene: proteoglycan↑	[104]
13	Emodin	*Rheum palmatum* L.	Intra-articular injection	ROMIBA	Cartilage erosion↓	–	[103]
14	Polydatin	*Polygonum cuspidatum*/	Intraperitoneal injection	MOMIBD	Synovial inflammation↓ Narrowing of joint space↓ Osteophyte↓ Cartilage erosion↓ Loss of high protein polysaccharide↓	–	[35]
15	*Panax quinquefolium* saponin	*Radix panacis* quinquefolii	Gavage	Rat OA model induced by ACLT combined with medial meniscal resection on the right knee joint	Destruction of articular cartilage↓	Apoptosis related factors: CHOP, caspase-3↓	[30]
16	Aucubin	Leaves of *Aucuba japonica*	Gavage	MOMIBD	Loss of proteoglycan↓ Hyaline cartilage thickness↑ Chondrocyte hypertrophy↓	–	[147]
17	Myricetin	Vegetables, tea	Gavage	MOMIBD	ECM of cartilage↑	Signal transduction pathways: p-AKT, Nrf2↑. NF-κB↓	[48]
18	Sinomenine	*Sinomenium acutum*	Intraperitoneal injections	MOMIBD	Articular cartilage thickness↑	–	[27]
19	Icariin	Epimedium	Intra-articular injection	Rat OA model induced by MIA	Cartilage erosion↓	Inflammatory factors: NLRP3, IL-1β, IL-18↓ Cartilage catabolic genes: MMP-1, MMP-13↓ Cartilage anabolic gene: collagen II↑	[196]
20	Sinapic acid	Spices, fruits, vegetables	Gavage	MOMIBD	Destruction of cartilage↓ Cartilage erosion↓ Loss of proteoglycan↓	Cartilage anabolic gene: collagen II↑ Cartilage catabolic gene: MMP-13↓ Signal transduction pathway: Nrf2↑	[26]
21	Peiminine	*Fritillaria thunbergii*	Intraperitoneal injection	MOMIBD	Osteophytes↓ Formation of calcified cartilage surfaces↓ Synovitis↓ Cartilage erosion↓	Signal transduction pathway: Nrf2↑	[52]
22	Quercetin	Fruits and vegetables	Intraperitoneal injection	MOMIBD	Cartilage degeneration↓ Chondrocyte apoptosis↓	level of oxidative stress biomarker:8-OHdG↓ Apoptosis related factor: caspase3↓	[138]
23	Loganin	Corni Fructus	Intra-articular injection	MOMIBD	Cartilage erosion↓ Loss of proteoglycan↓ Articular cartilage thickness↑ Osteophyte↓ Formation and calcified cartilage↓	Cartilage catabolic genes: MMP-3, MMP-13↓ Cartilage anabolic genes: Col10↓, Col2↑ Cell pyroptosis associated factor: caspase-1↓ Signal transduction pathway: NF-κB↓	[111]
24	Asiatic acid	*Centella asiatica*	Intra-articular injection	ROMIBA	Cartilage erosion↓ Loss of proteoglycan↓ Osteophyte↓	Chondrocyte hypertrophic markers: COL-X, MMP-13, Runx2↓ Chondrocyte fibrosis markers: COL-1, α-SMA↓ Signal transduction pathways: AMPK↑, PI3K, AKT↓	[143]
25	Morusin	*Morus australis*	Gavage	MOMIBD	Cartilage erosion↓ Destruction of cartilage↓	–	[141]
26	Salidroside	*Rhodiola rosea*	Oral administration	ROMIBA	Chondrocyte proliferation↑	Cartilage anabolic genes: Collagen II, Aggrecan↑ Cartilage catabolic gene: MMP-13↓ Signal transduction pathways: IκB-α, NF-κB↓	[109]
27	Astilbin	*Astilbe chinensis*	Gavage	Rat OA model	Destruction of cartilage↓ Cartilage layer↑ Loss of proteoglycan↓	Inflammatory factors: TNF-α, IL-6↓ Signal transduction pathways: AKT, PI3K↓	[74]
28	Moracin	Cortex Mori	Gavage	ROMIBA	Cartilage erosion↓ Destruction of cartilage	Cartilage anabolic genes: Collagen II↑ Signal transduction pathway: Nrf2↑	[45]
29	Myricitrin	*Myrica rubra*	Gavage	MOMIBD	Loss of proteoglycan↓ Cartilage erosion↓ Osteophytes↓	–	[49]
30	Morroniside	*Cornus officinalis*	Gavage	MOMIBD	Degeneration articular cartilage↓ Osteophyte↓	Inflammatory factors: Cox-2↓ Cartilage catabolic genes: MMP-3, MMP-13↓	[148]
31	Glycyrrhizin	Licorice root	Intra-articular injection	Rat OA model stimulated by ACLT and MMx	Joint edema↓	Inflammatory factors: IL-1β, IL-6, TNF-α, iNOS, PGE2↓ Cartilage catabolic genes: MMP-1, MMP-3, ADAMTS-5↓ Cartilage anabolic genes: aggrecan, Col2A1, Sox9↑, CTX-II↓ Signal transduction pathways: HMGB1, TLR4, p65, NF-κB↓	[43]
32	Baicalein	*Scutellaria baicalensis*	Intra-articular injection	RAT OA model stimulated by DMM	Sclerosis of subchondral bone↓ Synovial cells proliferation↓	Osteogenic marker genes: Alp, Runx2, OCN↓ Apoptosis related factors: caspase-3, Bax↑, Bcl-2↓	[139]
33	Quercetin	Fruits and vegetables	Intraperitoneal injection	ROMIBA	Degeneration and erosion of articular cartilage↓	Inflammatory factors:IL-18, NLRP3, TNF-α↓ Signal transduction pathway: IRAK1↓ Apoptosis related factor: caspase-3↓	[90]
34	Danshensu	*Salvia miltiorrhiza*	Intraperitoneal injection	MOMIBD	Destruction of cartilage surface↓ Cartilage erosion↓	–	[39]
35	Hyperoside	Hypericum	Intraperitoneal injection	MOMIBD	Loss of proteoglycan↓ Destruction of cartilage surface↓ Cartilage erosion↓	–	[110]
36	Curcumin	*Curcuma longa*	Intra-articular injection	Rat OA model stimulated by DMM	Synovial inflammation↓ Osteophyte↓ Subchondral bone plate thickening↓	Inflammatory factors: IL-6, IL-1β, TNF-α↓ Cartilage anabolic gene: type II collagen↑	[140]
37		*Curcuma longa*	Oral administration	MOMIBD	Chondrocyte proliferation↑	Signal transduction pathways: NF-κB, HIF-2α↓	[146]
38		Turmeric	Oral administration	MOMIBD	Loss of proteoglycan↓ Cartilage erosion↓ Synovitis↓ Subchondral plate thickness↓	Cartilage catabolic genes: MMP-13 and ADAMTS-5↓ Pro-inflammatory mediators: adipokines adipsin, leptin, adiponectin↑ Cartilage anabolic genes: type II collagen↑	[145]
39		Turmeric	Intraperitoneal injection	ROMIBA	Loss of chondrocytes↓ Loss of proteoglycan↓	Apoptosis related factors: CHOP, caspase-3↓ Cartilage anabolic genes: proteoglycan↑	[197]
40		*Curcuma longa*	Intra-articular injection	Rat OA model stimulated by ACLT and MMx	Synovial inflammation↓ Articular cartilage thickness↑ Chondrocyte apoptosis↓	Cartilage anabolic genes: collagen 2a1↑ Cartilage catabolic genes: MMP-3↓ Apoptosis related factors: cleaved caspase-3↓ Signal transduction pathways: VEGF, RUNX2, Hif-2α↓	[105]

## Rat OA models

### DMM-induced rat OA model

DMM, a commonly utilized model for OA, simulates key aspects of the disease including cartilage loss, osteophyte formation, subchondral hardening, and synovial inflammation. These NPs were administered to OA animals by oral administration, gavage, intraperitoneal injection, and intra-articular injection. (1) Intraperitoneal injection. In a rat DMM model, injection of α-Mangostin (10 mg/kg, once every 2 days), extracted from mangosteen, increased articular cartilage thickness and prevented the chondrocytes reduction and cartilage matrix degradation [46]. Quercetin, a widely found flavones in vegetables and fruits, had demonstrated protective effects cartilage degeneration in rat OA models [138]. (2) Oral administration. Oral administration of *Anthriscus sylvestris* leaf extract inhibited not only cartilage superficial destruction and cartilage erosion, but also proteoglycan depletion in a rat DMM model [104]. (3) Intra-articular injection. Baicalein (1 mg/week), another flavonoid derived from *Scutellaria baicalensis* Georgi, demonstrated beneficial effects in a rat OA model via inhibiting pre-osteoblast differentiation, proliferation, synovial cell proliferation, and the expression of osteogenic and increasing apoptotic markers of pre-osteoblasts [139]. In DMM rats, injection of curcumin monoglucuronide alleviated synovial inflammation and protected cartilage integrity [140]. (4) Gavage. Morusin (40 mg/kg, once every two days), a prenylated flavonoid from *Morus australis* root bark, inhibited cartilage erosion and destruction in a rat OA model [141]. Overall, using DMM-induced rat OA models, these findings from different studies confirmed the potential of NPs such as α-Mangostin, *Anthriscus sylvestris* extract, curcumin monoglucuronide, quercetin, morusin, and baicalein in attenuating OA-associated pathological changes.

### ACLT-induced rat OA model

ACLT is another common method used in the studies of OA progression. (1) Intraperitoneal injection. Injection of quercetin alleviated OA progress by decreasing the production of inflammatory markers, such as IL-1β, IL-18, and TNF-α, as well as reduction of IRAK1, NLRP3, and caspase-3 expression [90]. (2) Oral administration. In a rat OA model, oral administration of salidroside (12.5 mg/kg or 25 mg/kg once a day), a bioactive component isolated from *Rhodiola rosea*, also exhibited beneficial effects by modulating immune response, including upregulating CD4+ IL-10+ cells and inhibiting NF-κB activation [109]. (3) Intra-articular injection. In an ACLT-stimulated rat OA model, the injection of Ginsenoside Rb1, Chinese herb Panax ginseng extract, exhibited chondroprotective properties by inhibiting MMP-13 expression through inhibiting of the Notch signaling pathway [142]. Emodin is a natural anthraquinone that shown to inhibit cartilage erosion and chondrocyte loss in an ACLT-stimulated OA model [103]. In an ACLT and MMx induced, rat OA model, the injection of chemically modified curcumin (20 μM or 40 μM, once a week) alleviated synovial inflammation, increased articular cartilage thickness, and inhibited chondrocyte apoptosis by suppressing the expressions of MMP-3, cleaved caspase-3, VEGF, RUNX2, and HIF-2α, promoted collagen generation, and inhibiting NF-KB pathway [105]. Asiatic acid, a pentacyclic triterpene existed in *Centella asiatica*, reduced chondrocyte hypertrophy and fibrosis through promoting AMPK phosphorylation and inhibiting PI3K and AKT phosphorylation in a rat OA model [143]. Glycyrrhizin, a major constituent of licoriceduced joint edema and regulated the gene expression of cartilage degradation-related biomarkers which were related to TLR4/NF-κB and HMGB1 signaling pathway [43]. (4) Gavage. Ginseng, a widely used herbal medicine, contains ginsenoside Rg1 as one of its main active components. Ginsenoside Rg1 could alleviate cartilage erosion, joint surface wear, and reduced expression of MMP-13 in a rat OA model [144]. It inhibited the expression of related inflammatory mediators, such as PGE2 and COX-2, while promoting the production of type II collagen and aggrecan [144]. *Panax quinquefolium* saponin, the major active compound of *Radix panacis* quinquefolii, demonstrated protective effects in a rat OA model by reducing the levels of CHOP and caspase-3, markers associated with chondrocyte apoptosis, and inhibiting the process of articular cartilage morphology and structure destruction [30]. Moracin, a kind of flavonoid compound which extracted from Cortex Mori, was found to have beneficial effects in an ACLT rat OA model. Administration of moracin promoted the expression of collagen II and Nrf2, alleviated superficial cartilage damage, extracellular matrix loss, and cartilage erosion [45]. In summary, using ACTL-induced rat OA models, these findings have confirmed the potential of various NPs in alleviation of OA progression. Ginsenosides, including GRb1 and Rg1, showed protective effects by modulating protein expressions, promoting collagen expression, and inhibiting inflammatory mediators. Emodin, *Panax quinquefolium* saponin, curcumin, and chemically modified curcumin exhibited cartilage-protective properties through apoptosis inhibition, collagen promotion, and inflammation regulation. Asiatic acid, salidroside, moracin, glycyrrhizin, and quercetin demonstrated beneficial effects by modulating chondrocyte activities, reducing cartilage damage, and regulating inflammatory responses. These findings provide valuable insights into potential therapeutic strategies for OA management.

## Mouse OA models

DMM model is commonly employed in mice to establish OA model by disrupting joint stability. NPs were administered to DMM-induced OA mice by intraperitoneal injection, oral administration, intra-articular injection, and gavage. (1) Intraperitoneal injection. Xu et al. discovered that injection of danshensu inhibited cartilage surface destruction, erosion, and chondrocyte loss in DMM-induced OA mice [39]. Salvianolic acid B (25 mg/kg, once every 2 days), a kind of water-soluble polyphenolic acid, exhibited a slowing effect on cartilage matrix and articular cartilage destruction in DMM-induced mice [40]. Polydatin (100 mg/kg, once a day), a kind of natural resveratrol glucoside which derived from *Polygonum cuspidatum*, alleviated synovitis and protected cartilage in a DMM-induced mice OA model [35]. Sinomenine, a monomeric component purified from *Sinomenium acutum*, could inhibit articular cartilage thinning and cartilage matrix degradation in a DMM mice model [27]. Peiminine, a native compound derived from *Fritillaria thunbergii*, alleviated synovitis and delayed OA progression by activating Nrf2 nuclear translocation [52]. Hyperoside, a bioactive flavonoid glycoside found in epimedium, hypericum, and perforatum, decreased proteoglycan loss and inhibited superficial cartilage destruction and erosion in DMM mice [110]. (2) Oral administration. Curcumin demonstrated its ability to alleviate synovitis and cartilage damage in a mouse OA model via inhibiting the production of MMP-13, ADAMTS-5, adiponectin, adipokines adipsin, and leptin in the infrapatellar fat pad [145]. Wang et al. confirmed that oral curcumin could promote chondrocyte proliferation and suppressing the activation of the NF-κB/HIF-2α pathway in a mice OA model [146]. (3) Intra-articular injection. In a DMM mice model, Loganin injection inhibited hypocellularity, cartilage superficial destruction, proteoglycan loss, decreased calcified cartilage partially through reducing p-IκB protein level of cartilage [111]. (4) Gavage. Piceatannol, a hydroxystilbene found in various foods, displayed protective effects on articular cartilage by inhibiting cartilage calcification, cartilage surface destruction, synovial inflammation, and osteophyte generation a mouse OA model [50]. Aucubin, a naturally occurring product found in lots of plants, exhibited protective effects in a mouse OA model by reducing proteoglycan loss, cartilage fibrillation, erosion, and chondrocyte disordering and hypertrophy [147]. Myricetin, a naturally occurring flavanol derived from vegetables and fruits, protected cartilage ECM and prevented cartilage damage by the PI3K/Akt-mediated Nrf2/HO-1 signaling pathway DMM-induced mice [48]. Using DMM-induced mice OA model, Sinapic acid exhibited protective effects through suppressing proteoglycan loss, erosion, hypocellularity and cartilage destruction [26]. Morroniside, an iridoid glycoside, exhibited effects of anti-inflammatory and cartilage protection by reducing the COX-2, MMP-3, and MMP-13 expression in a mouse OA model [148]. In summary, several NPs exhibited promising anti-OA effects in DMM-induced mice, including the reduction of synovitis and improvement of cartilage mass by regulating several signaling pathways.

## Other OA models

DMM and ACLT-induced rats and mice OA models are common animal methods in the studies of NPs for OA treatment. In addition, there were other OA animal models that have been used in these fields. Oral administration of astilbin (3 mg/kg, once a day) in a papain-induced OA rat model inhibited cartilage destruction and proteoglycan loss, promoted cell number and cartilage layer thickness by PI3K/AKT pathway [74]. In a MIA induced rat OA model, oral administration of Fuzi demonstrated positive effects on prevention of joint injury, and inhibition of bone density decline [149]. In a rat OA model induced by modified hulth technique, Ping et al. had confirmed that oral administration of *Eucommia ulmoides* Oliv. Bark (EU) aqueous extract inhibited joint degeneration and mitigated articular cartilage destruction via suppressing MMP-3 secretion and downregulating expression of p-AKT in chondrocytes [150]. Besides rats and mice, rabbits are another alternative to mimic OA progression. Chen et al. found that injection of chlorogenic acid (20 μM, once a week) could protect rabbit articular cartilage from degradation by down-regulating of MMP-1, MMP-3, and MMP-13 and up-regulating TIMP-1 expression in an ACLT-induced rabbit OA model [102]. In a rabbit OA model induced by ACLT, oral administration of pomegranate fruit extract effectively reduced cartilage destruction [151]. In addition, it promoted the gene expression of COL2A1 and ACAN partially through inhibiting apoptosis-related markers, such as PARP P85 and caspase-3 [151]. Using rabbits OA model, the authors found that pomegranate extract are helpful in preserving cartilage health of OA by distinct signaling pathways. More large animal models, such as goat, pig, and monkey, are needed to explore the bioeffects and mechanisms of NPs in the treatment of OA. It is known from the above studies that there are many types of NPs with different ways of administration, so finding a way to assess the optimal dosage of the specific NPs is necessity for safety and tolerability of NPs in OA patients.

## Clinical trials of NPs for OA patients

Numerous clinical trials have been conducted to investigate the clinical effects of various natural drugs in the treatment of OA (Table 2). These trials have primarily focused on patients with knee OA, but also encompassed individuals with hip and hand OA.

**Table 2:** Clinical trials of NPs in the treatment of OA

List	Compound	Source	OA-affected joint	Route of administration	Number	The research methods	Clinical effect	Reference
1	Chicory root extract	Chicory root	Hip and knee	Oral administration	40 patients	Phase1, placebo-controlled, double blind, dose-escalating study	Improvement in hip or knee function, stiffness, pain and other symptoms	[162]
2	GLM	*Perna canaliculus*	Knee	Oral administration	21 patients	An open label, single group allocation study	Knee pain, stiffness, and mobility improved significantly	[163]
3	Sphaeralcea angustifolia extract	*Sphaeralcea angustifolia* Cavanilles&Don	Hand	Topical administration of a gel	130 patients	A double-blind, randomized study controlled	Hand joint pain, inflammation, joint stiffness and motor function improved	[164]
4	HT-20	Olive leaves	Knee	Oral administration	25 patients	A double-blind placebo-controlled clinical trial	The painful symptoms of the knee were relieved	[166]
5	Artrim	Artemisia Annua	Hip and knee	Oral administration	42 patients	A pilot randomized, placebo-controlled clinical trial	The pain in the hip and knee OA was significantly improved	[169]
6	BioLex® -GLM extract	*Perna canaliculus*	Hip and knee	Oral administration	80 patients	A randomized double-blind placebo-controlled trial	The hip and knee pain was not relieved. The joint stiffness was reduced. The dose of acetaminophen used in OA patients could be reduced	[170]
7	Colchicine	*Colchicum autumnale*	Knee	Oral administration	109 patients	A double-blind, placebo-controlled, randomized trial	The markers of high bone turnover and inflammation in the knee OA were reduced. Symptoms were not reduced	[171]
8	Deer bone extract	Deer bone	Knee	Oral administration	50 patients	A randomized, double-Blind, placebo-controlled trial	The pain and stiffness in the OA of the knee were relieved. Joint function was improved	[168]
9	Green tea extract	Green tea	Knee	Oral administration	50 patients	A randomized open-label active-controlled clinical trial	Pain and stiffness of knee OA was relieved Joint function was improved	[172]
10	*Cucumis sativus* extract	Cucumber	Knee	Oral administration	122 patients	A randomized, double-blind, parallel-group clinical trial	Effective relief of pain, stiffness and function in knee OA	[165]
11	GCWB106	*Chrysanthemum zawadskii* var. latilobum	Knee	Oral administration	121 patients	A 12-week randomized, double-blind, placebo-controlled study	The OA pain in the knee was reduced. Knee function improved	[177]
12	NR-INF-02	Rhizome of *Curcuma longa*	Knee	Oral administration	120 patients	A randomized, single blind, placebo-controlled trial	Knee pain was reduced	[159]
13	Curcuminoid	*Curcuma longa*	Knee	Oral administration	53 patients	A pilot randomized double-blind placebo-control parallel-group clinical trial	Arthritis pain in the knee was significantly reduced. Knee function improved	[156]
14	Curcumin	*Curcuma longa*	Knee	Oral administration	160 patients	A randomized, double-blind, placebo-controlled trial	Knee function, stiffness, and pain improved significantly	[155]
15	Curcuminoid	*Curcuma longa*	Knee	Oral administration	797 patients	A systematic review and meta-analysis of randomized clinical trials	Mild improvement of knee pain and function	[157]
16	Bio-optimized *Curcuma longa* extract	*Curcuma longa*	Knee	Oral administration	150 patients	A prospective, randomized, 3-month, double-blind, multicenter, three-group, placebo-controlled trial	The OA pain in the knee was reduced	[158]
17	*Curcuma longa* extract	*Curcuma longa*	Knee	Oral administration	797 patients	A 12-week, single-center, randomized, placebo-controlled clinical trial	Symptoms of pain in the knee improved	[160]
18	*Curcuma longa* extract	*Curcuma longa*	Knee	Oral administration	1810 patients	A systematic review and meta-analysis of randomised controlled trials	Knee pain was significantly reduced Joint function was improved	[161]

## Curcumin in OA clinical trials

Notably, curcumin has been the subject of numerous clinical trials exploring its potential in OA treatment. Curcumin is a component of the traditional medicine *Curcuma longa* [152]. Curcumin had protective effects on various diseases, including cancer, diabetes and inflammatory diseases [153, 154]. Notably, several studies have reported the positive efficacy of curcumin in the treatment of knee OA. Srivastava et al. found that oral curcumin improved knee pain, stiffness, and function, while reduced levels of inflammatory markers and biomarkers of oxidative stress in blood, such as IL-1β and ROS and malondialdehyde [155]. Panahi et al. demonstrated that oral curcuminoid significantly alleviated visual analogue scale (VAS) score and improved function of knee, but did not improve joint stiffness of OA patients [156]. Similarly, Onakpoya et al. conducted experiments on 797 patients with knee OA and found that curcuminoids significantly reduced VAS score and improved quality of life in OA patients [157]. In a prospective, randomized, double-blind, multicenter trial, Henrotin et al. found that curcuma longa extract reduced knee OA pain and down-regulated the biomarker of cartilage degradation, serum sColl2-1 [158]. Madhu et al. conducted a clinical trial in 120 patients with knee OA and found that oral administration of curcuma longa extract relieved pain and improved knee function for patients with primary painful knee OA [159]. Wang et al. conducted a clinical trial and found that curcuma longa extract reduced pain in knee OA patients and but did not affect cartilage composition and effusion–synovitis in knee [160]. Furthermore, a meta-analysis confirmed that the oral administration of curcuma longa extract significantly reduced knee pain and improves joint function of OA patients [161]. Collectively, these studies suggest the potential of curcumin and its derivatives in alleviating symptoms and function of knee OA patients, especially for pain relief. More multicenter trials are needed to confirm those clinical benefits of curcumin for OA patients.

## Other NPs in OA clinical trials

In addition to curcumin, the potential therapeutic effects of other NPs have been investigated in clinical trials for OA patients. For instance, in a placebo-controlled, double blind, dose-escalating trial, chicory, a perennial herb in the Asteraceae family, reduced at least 20% improvement in stiffness and pain of OA patients [162]. In an open-label study for knee OA, green-lipped mussel (GLM) extract (3,000 mg/day) significant reduced Western Ontario and McMaster Universities Osteoarthritis Index (WOMAC) score and the Lequesne algofunctional index, showing improvement in knee pain, stiffness and mobility [163]. In this clinical trial, adverse reactions were mainly gastrointestinal symptoms, including reflux in 1 case, abdominal pain, and diarrhea in 1 case, and gout in 2 cases [163]. In addition, *Sphaeralcea angustifolia* reduced pain, inflammation, and stiffness of hand joints of 130 patients with OA, and none of the patients experienced adverse reactions [164]. Short-term or long-term oral administration of cucumis sativus extract effectively reduced the WOMAC and VAS scores in a randomized controlled trial and no adverse reactions were reported [165]. Hydroxytyrosol, which is mainly found in olive leaves, was evaluated by Takeda et al. in a double-blind clinical trial for the treatment of knee OA and the results showed that oral administration of hydroxytyrosol relieved Japanese Orthopedic Association score (pain measurement index) [166]. In a 12-week randomized, double-blind, placebo-controlled clinical study, GCWB106 (600 mg/day), a formulated extract from *Chrysanthemum zawadskii* var. latilobum, significant alleviated WOMAC and VAS scores of OA patients and no adverse reactions were reported [167]. Shin et al. found that oral administration of deer bone extract at a daily dose of 550 mg for 12 weeks reduced knee pain and stiffness and improved joint function in mild to moderate OA [168]. A 12 weeks’ study was conducted on OA patients, which revealed that the oral consumption of Artemisia annua extract notably relieved WOMAC pain [169]. In this study, the most common adverse event was gastrointestinal reflux [169]. However, there are some negative results in the effects of specific NPs on OA symptoms. Oral BioLex®-GLM extract, derived from *Perna canaliculus*, did not provide relief for hip and knee pain but reduced paracetamol intake compared to the placebo group [170]. Adverse events occurred in 1 patient with abdominal pain in the treatment group in this trail [170]. Besides, in a randomized controlled trial, oral colchicine reduced inflammation and high bone-turnover biomarkers but did not relieve knee OA symptoms like WOMAC score [171]. However, mild diarrhea adverse events occurred in both the treatment group and the placebo group [171]. Green tea extract was investigated in a clinical trial, indicating its clinical effects of improving knee pain and joint function, but did not significantly improve stiffness symptoms [172]. No adverse events were observed in the green tea group [172]. Preliminary clinical studies have indicated the efficacy of these NPs in relieving OA symptoms with limited side effects, suggesting NPs’ potential as important for OA treatment in the future. Further optimization and exploration of NPs in clinical trials are warranted to enhance their clinical applications for early intervention and management of OA.

## Conclusion and outlook

Overall, specific NPs ameliorate the progression of OA via inhibiting inflammation, modulating anabolic and catabolic processes, and preventing cell death. Mechanistically, NPs inhibited inflammation mainly by suppressing the expression and phosphorylation of IκB and p65, increasing p-IκB degradation, promoting p65 nuclear translocation, phosphorylation of MAPKs, PI3K, AKT and mTOR, and increasing SIRT1 activation during the treatment of OA. In addition, NPs could promote the anabolism and suppress the catabolism of chondrocyte in OA treatment mainly by inhibiting NF-κB, MAPKs and PI3K-AKT pathways, and activation of SIRT1. Moreover, NPs reduced chondrocyte apoptosis by inhibiting the expression of apoptotic proteins like Bax and Bad, and increasing anti-apoptotic proteins like Bcl-XL and Bcl2. Using several routes of NPs’ administration in different OA animal models, such as intraperitoneal injection, oral administration, intra-articular injection and gavage, many studies have uncovered the bioeffects and mechanisms of NPs on the OA. In the clinical trials of OA patients, NPs have been exhibited the ability to alleviate clinical symptoms, such as pain and joint stiffness, and improve joint function in OA progression.

Currently, several first-line drugs are recommended in the treatment of OA, such as nonsteroidal anti-inflammatory drugs (NSAIDs), steroids, central analgesics, and chondroitin [173]. Compared with those pharmacologic approaches, NPs have several advantages. Long-term use of NSAIDs or steroids increases the risk of gastrointestinal disorders and cardiovascular complications other diseases [174], while there were limited side effects of NPs reported in the clinical trials of OA treatment. NPs have less drug tolerance and dependence, which has the possibility of replacing central analgesics. Yu et al. found that huperzine A alleviated neuropathic pain via inhibition on acetylcholinesterase and NMDA receptors [175]. Compared with glucosamine-chondroitin, lower doses of the NPs had better effects in the treatment of osteoarthritis, including relief of knee pain, stiffness, and physical functions related to OA [165]. In addition, NPs have more abundant sources and multiple components with multiple targets. Nonetheless, there are some disadvantages of NPs for OA treatment. For example, most NPs have complex chemical composition and low bioavailability. Besides, the application of NPs is controversial due to their unclear mechanisms of bioeffects on different tissue or cells. Thus, more clinical and fundamental studies are needed to verify therapeutic effect and mechanisms of NPs on OA treatment.

More advanced biomedical technology and methods are powerful tools in studies of NPs for OA patients. For example, artificial intelligence or machine learning could be used to efficiently screening of potential NP drugs with optimal therapeutic effects and dose–effect relationship of these drugs [176, 177]. Single-cell genomics could be a powerful tool to explore the pathological changes of OA after NPs’ treatment [178]. Besides, animals with genetically engineered change also can be used to explore the potential NPs’ targets and detailed mechanisms of NPs’ treatment. Wan et al. found that HHQ16, a novel small molecule derived from astragaloside IV, could improve cardiac function and lnc9456 was bona fide target of HHQ16 via transgenic mice [179]. Using cryo-electron microscopy (cryo-EM), Liao et al. recently uncovered high-resolution cryo-EM structure the cannabinoid receptor 1-arrestin complex, facilitating potential drug design targeting cannabinoid receptor 1 [180]. The utilization of NPs exhibits significant promise in the clinical management of OA. More underlying bioeffects and mechanisms of NPs on OA are needed to be explored via advanced technology and methods.

**Fig. 5:**
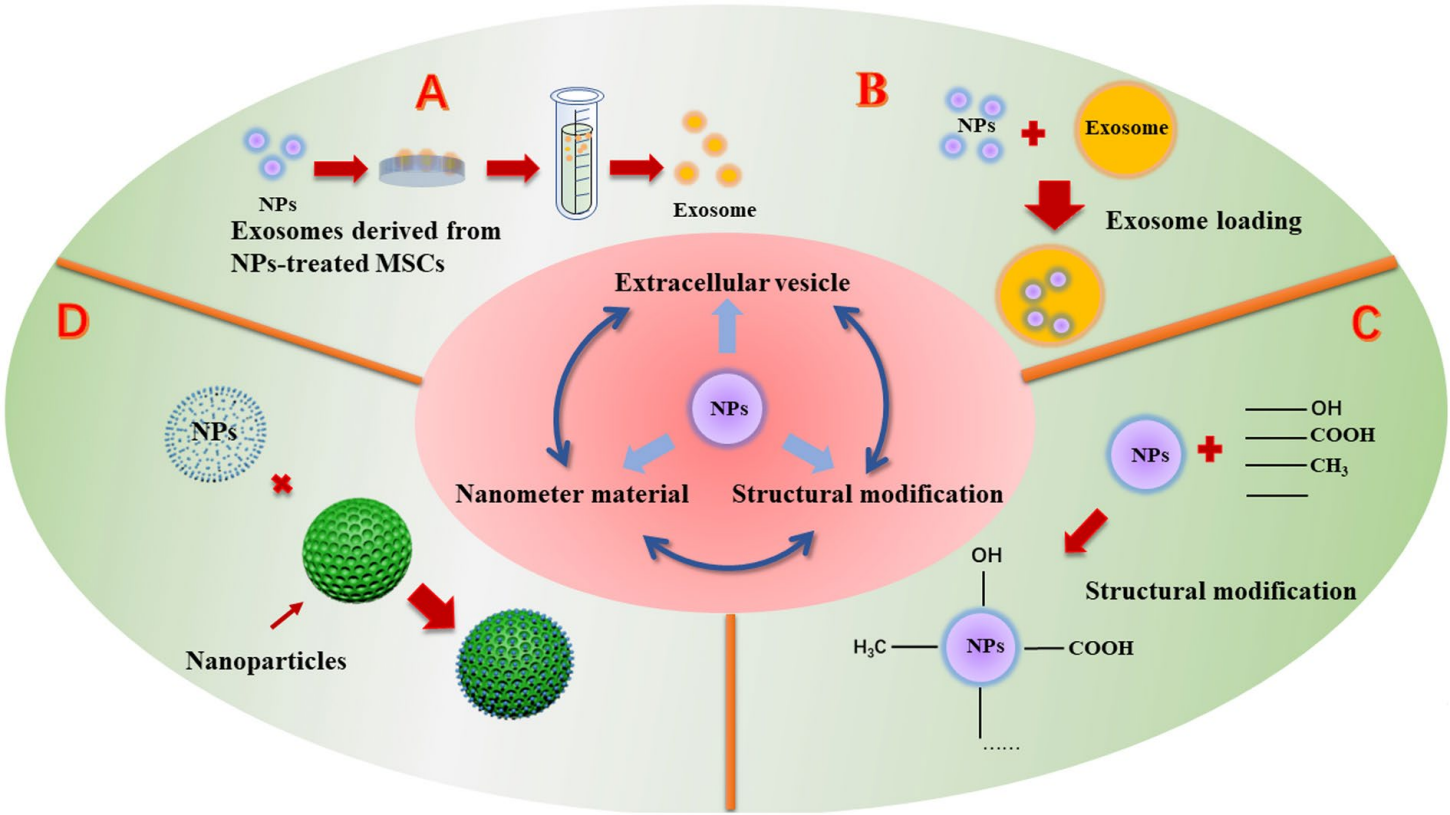
Potential new application strategies of NPs for the OA treatment. **A** Exosomes derived from MSCs treated with NPs hold significant potential for the treatment of OA. **B** Exosomes, membrane-bound vesicles secreted by various cells, serve as extracellular vehicles. Utilizing exosomes as delivery vectors for NPs or exosome-loading method harnesses the potential of exosomes to transport NPs and presents a potentially viable therapeutic model for the management of OA. **C** Structural modification of NPs enables the development of novel drugs with enhanced efficacy. During the treatment of OA, the therapeutic potential of NPs can be improved through structural modifications. **D** Hybrid nanospheres possess outstanding pH/thermal sensitivity, superior drug release profiles, and excellent biocompatibility. NPs based on nanomaterials hold great promise for enhancing efficacy for OA treatment

Furthermore, there are various strategies that can be applied to improve and optimize bioavailability of NPs in OA treatment (Fig. 5). Extracellular vesicles consist of membrane-bound structures, including three categories: exosomes, microvesicles, and apoptotic bodies [181]. Recently, it has been found that extracellular vesicles, especially exosomes, are good choices as drug carriers. Exosomes, which are extracellular vesicles enclosed by a membrane and released through various cells, play crucial roles in intercellular communication, both in physiological and pathological contexts. Due to their low immunogenicity and high stability, exosomes hold significant potential as oral drug delivery vectors [182]. Similarly, exosomes derived from curcumin-treated MSCs have demonstrated the ability to suppress chondrocyte apoptosis and ameliorate the severity of OA [183]. Exosomes derived from mesenchymal stem cells of the infrapatellar fat pad have shown the ability to protect chondrocytes in mice with OA and mitigate the progression of OA [184]. Further exploration of the therapeutic efficacy of exosomes derived from specific stem cells treated with NPs is warranted. In addition, the synergistic bioeffects and mechanisms between extracellular vesicles and NPs on OA are needed. What’s more, the loading of NPs by exosomes can promote the efficient enrichment of NPs in local tissues, and it is also expected to achieve cell-specific targeted delivery, which needs further attention in future study.

Structural modification of NPs is another important strategy to improve their therapeutic properties, especially for novel drug discovery [185]. Chemically modified curcumin has the potential to improve drug bioavailability and exhibit enhanced therapeutic efficacy for OA [105]. Arctigenin (ARG), a lignan-class compound derived from *Arctium lappa* L., has limited solubility and bioavailability [186]. Cai et al. synthesized ARG amino acid derivatives with improved solubility and nitrite clearance, exhibiting higher tumor inhibition rates than original ARG [187]. Structural modification of NPs holds promise not only for OA but also for other diseases such as tumors and immune system disorders. Modified NPs have the potential to reduce side effects and improve therapeutic efficacy.

Biomaterials also have potential advantages in improving the therapeutic effectiveness of NPs. Biomimetic injectable hydrogel microspheres have emerged as a highly promising drug delivery system for the effective treatment of OA. These microspheres can effectively encapsulate diclofenac sodium, enhancing lubrication and enabling sustained drug release [188]. Ji et al. developed an innovative drug delivery system by employing porous chitosan microspheres and hydroxypropyl chitine hydrogels, which can not only regulate macrophage polarization but also promote chondrocyte regeneration [189]. By achieving sustained drug release, this system has the potential to reduce the frequency of drug administration, resulting in a better treatment experience for patients. In addition to synthetic carriers, human cells such as red blood cells (RBCs), neutrophils, and lymphocytes have shown potential as drug carriers. These cells offer advantages such as enhanced therapeutic efficacy and targeted delivery, making them a promising approach for drug delivery [190]. RBCs, with their biconcave shape and non-nuclear structure, have the capacity to carry a larger payload of drugs while reducing toxic side effects [191]. Bonnie Huang et al. utilized autologous lymphocytes as carriers to target nanoparticles to lymphatic tissue sites in the treatment of lymphoma, thereby enhancing therapeutic efficacy without increasing adverse side effects [192]. Theoretically, NPs can serve as carriers for human cells, allowing for precise delivery to specific tissues such as articular cartilage and synovial membrane. Moreover, hybrid nanospheres exhibit favorable pH and thermal sensitivity, exceptional drug release profiles, and biocompatibility, making them highly promising for applications in anticancer therapy [193]. Their ability to sense temperature and pH opens up new possibilities for the management of OA and other ailments, representing a valuable research direction. By incorporating temperature and pH-sensitive components, drug delivery systems can respond to variations in inflammatory markers including IL-6, PGE2, and IL-1β, leading to improved therapeutic outcomes for OA. NPs combine with these new biomaterials and carriers hold promise for achieving improved therapeutic effects with fewer side effects, higher stability and faster responsiveness, not only in the treatment of OA but also other diseases.

Physical therapy, including exercise, ultrasound, and electrotherapy, is a first-line clinical treatment for OA. Lubrano et al. reviewed the synergistic effect of rehabilitation therapy combined with TNFα inhibitor therapy on ankylosing spondylitis, showing that the combination was more effective than TNFα inhibitors alone [194]. In a blinded, prospective, randomized, controlled study by Saccomanno et al., the effectiveness of intra-articular hyaluronic acid injections and individualized rehabilitation programs for knee OA, the combined treatment showed the greatest pain relief [195]. Combining NPs with physical therapy holds the potential for unexpected therapeutic benefits and fewer side effects, and further investigation is required to understand and evaluate their synergistic effects.

In brief, NPs is a potentially effective conservative strategy for OA patients. The therapeutic efficacy of different types of NPs has been confirmed in OA treatment. More advanced technology and methods, including but not limited to artificial intelligence, single-cell genomics, transgenic animals, cryo-EM, etc. could be used in the study of this field. In addition, structural modification of NPs, loading of NPs by exosomes, and combination of NPs with new biomaterials are potential strategies for the future study. Moreover, further studies should focus on the precise bioeffects, underlying mechanisms, dose–effect relationship, safety and tolerability of clinical usage for NPs to maximize NPs’ benefits for OA patients.

## Data Availability

Not applicable.
